# Efficacy of a novel chewable tablet (Credelio Quattro^™^) containing lotilaner, moxidectin, praziquantel, and pyrantel against *Toxocara canis* and *Toxascaris leonina* infections in dogs

**DOI:** 10.1186/s13071-025-06968-9

**Published:** 2025-08-20

**Authors:** Samuel Charles, Scott Wiseman, Xinshuo Wang, Molly D. Savadelis, Craig R. Reinemeyer, Imad Bouzaidi Cheikhi, Carin Rautenbach, Lisa Young

**Affiliations:** 1 Elanco Animal Health, Innovation Way, Greenfield, IN 46140 USA; 2 Elanco Animal Health, Bartley Way, Hook, RG27 9XA UK; 3 East Tennessee Clinical Research, Rockwood, TN USA; 4 Clinvet, Mohammedia, Morocco; 5 Clinvet, Bloemfontein, South Africa

**Keywords:** Credelio Quattro, Roundworms, *Toxocaracanis*, *Toxascarisleonina*

## Abstract

**Background:**

Roundworms such as *Toxascaris leonina* and *Toxocara canis* are routinely diagnosed in dogs globally, especially in dogs 6 months of age or younger. *Toxocara canis* is zoonotic, can cause significant disease in dogs, and is the causative agent of toxocariasis in humans. To protect both animal and human health, it is imperative that *Toxocara canis* infections are effectively treated and controlled to minimize the risk of transmission. The following studies were performed to demonstrate the effectiveness and safety of a novel, combination chewable tablet (Credelio Quattro^™^) containing the minimum effective dosages of lotilaner (20.0 mg/kg), moxidectin (0.02 mg/kg), praziquantel (5.0 mg/kg), and pyrantel (5.0 mg/kg) for the treatment and control of *T. canis* and *T. leonina* infections in dogs.

**Methods:**

Six well-controlled studies were performed. Two studies each evaluated Credelio Quattro against immature adult *T. canis*, adult *T. canis*, and adult *T. leonina* infections. Post-treatment efficacy was calculated from necropsy worm counts, and fecal egg count reduction was determined 10 days post-treatment in studies evaluating experimentally induced or naturally acquired adult infections.

**Results:**

Credelio Quattro was safe and ≥ 97.9% effective against immature adult stages and ≥ 97.0% effective against adult stages of induced and natural *T. canis* infections in dogs. After treatment with Credelio Quattro, fecal egg counts were reduced by ≥ 98.8% in *T. canis*-infected dogs. In both experimentally induced and naturally acquired adult *T. leonina* infections in dogs, Credelio Quattro was safe and 100% effective in eliminating adult worms and provided 100% reduction in fecal egg counts post-treatment. The most common adverse events reported included digestive tract disorders such as diarrhea, mucus and/or blood in feces, vomiting, and expelled ascarid worms, which occurred in both control- and treated-groups.

**Conclusions:**

These laboratory studies confirm the effectiveness and safety of a single dose of Credelio Quattro, administered at the minimum dosages of 20 mg/kg lotilaner, 0.02 mg/kg moxidectin, 5 mg/kg praziquantel, and 5 mg/kg pyrantel, for the treatment and control of immature adult and adult *T. canis* and adult *T. leonina* in dogs.

**Graphical abstract:**

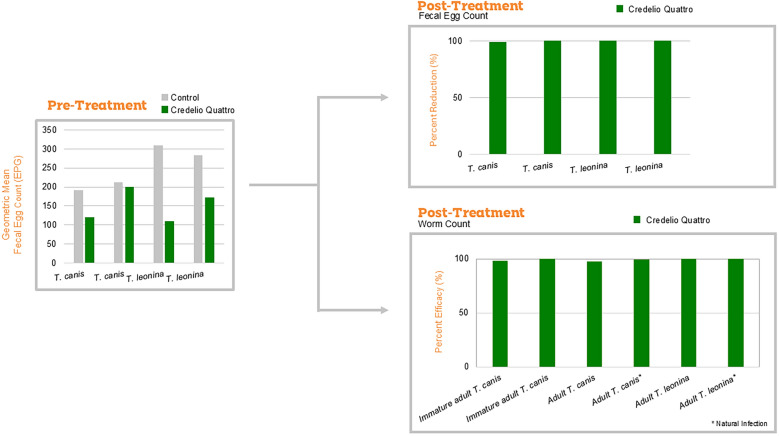

## Background

Roundworm infections with *Toxocara canis* and *Toxascaris leonina* in dogs are diagnosed routinely around the globe. Global prevalence of *T. leonina* was recently estimated to be 2.9% in dogs through a quantitative analysis of 135 published studies, with stray dogs testing significantly higher than pets [1]. Within this analysis, the North American prevalence was estimated to be 2.0%, with a 95% confidence interval of 1.1–3.2% [1]. While infection rates may be higher in strays or primarily outdoor pets with access to prey, ascarid infections are common in all dogs. A study evaluating 3006 fecal samples collected from pets visiting dog parks in 30 major metropolitan areas throughout the USA reported that 622 (20.7%) dogs tested positive for the presence of gastrointestinal parasites with detection of ascarid eggs in 17 (0.6%) samples [2]. Limited national studies have been performed to evaluate the prevalence of *T. canis* in the USA. The most recently conducted survey (2008–2009) evaluated 2600 dogs in animal shelters and determined a prevalence of 14.2% [3, 4]. The Companion Animal Parasite Council (CAPC) generates prevalence maps utilizing data from Antech Diagnostics, IDEXX Laboratories, and Zoetis Diagnostics that provide more recent information on ascarid prevalence in the USA and Canada. For 2024, CAPC reported the national prevalence of roundworms (*T. canis* and *T. leonina*) at 1.44% in the USA [5]. These reported data only represent results from animals receiving veterinary care and according to CAPC, are estimated to represent less than 30% of total cases in a geographical region.

*Toxocara canis* and *T. leonina* can be transmitted to dogs through the ingestion of larvated eggs from the environment or ingestion of infected paratenic hosts, such as rodents, containing larvae in the tissues [6, 7]. Once shed in the environment, *T. canis* eggs can take up to 2–4 weeks and *T. leonina* up to one week to develop to the infective stage, depending on environmental conditions [8–10]. Due to the thick outer shell, roundworm eggs can survive many years in more harsh environmental conditions as compared with other gastrointestinal nematode species, thereby enabling roundworm eggs to remain infective for long periods of time [11]. After ingestion of larvated eggs, *T. canis* larvae migrate to the liver and lungs, and then up the mucociliary apparatus where the larvae are then swallowed. If transmission occurs through ingestion of infected vertebrates, there is no larval tissue migration [12]. Through both routes of infection, sexually mature adult roundworms develop in the small intestine, and nonembryonated eggs are shed in the feces of infected animals. In adult dogs, many ingested roundworm larvae arrest in somatic tissues during migration and are then activated during pregnancy, infecting puppies in utero transplacentally and, to a lesser degree, through transmammary transmission while nursing [12]. Unlike *T. canis*, transmammary and transplacental transmission has not been documented to occur with *T. leonina*, nor do larvae migrate outside of the gastrointestinal tract of the definitive host [7, 12, 13].

Much of the pathology observed in roundworm infections in dogs is attributed to the presence of adult worms in the small intestine, with clinical disease more commonly associated with *T. canis* infections [3]. The most common clinical signs of ascarid infection in dogs include vomiting, diarrhea, failure to gain weight, and presentation of a pot-bellied appearance [14]. More rarely, with extremely large adult worm burdens, the small intestine can become impacted, leading to potential intestinal rupture and septic peritonitis. Infection in humans with *T. canis* can occur through fecal–oral contamination with ingestion of larvated eggs. Toxocariasis can be asymptomatic or induce visceral larva migrans, ocular larva migrans, or neurotoxocariasis [3, 15]. Although only *T. canis* is considered zoonotic, *T. leonina* has been hypothesized to infect humans rarely, with an unidentified *Toxascaris* spp. causing disease in one documented case [16, 17].

CAPC in the USA currently recommends that veterinarians test all dogs for the presence of gastrointestinal parasites by centrifugal fecal flotation at least four times in puppies less than 1 year old and then at least two times annually thereafter [18]. In addition, the utilization of parasite fecal antigen diagnostics or polymerase chain reaction (PCR) testing is recommended [18] to assist in accurately diagnosing ascarid infections with limited egg shedding. Due to the high occurrence of transplacental transmission with *T. canis* and its zoonotic potential, CAPC recommends all puppies be dewormed every 2 weeks starting at 2 weeks of age, until approximately 8 weeks of age, and then monthly thereafter with an appropriately labeled product against ascarids [18]. Efficacy of anthelmintic drugs should be monitored by centrifugal fecal examination post-treatment. In the USA, there are currently five drugs labeled for the treatment of roundworms in dogs by the US Food and Drug Administration (FDA): fenbendazole, moxidectin, febantel, pyrantel, and milbemycin oxime.

The objective of the studies presented here was to evaluate the efficacy and safety of a novel, oral chewable tablet containing lotilaner, moxidectin, praziquantel, and pyrantel (Credelio Quattro, Elanco Animal Health, Greenfield, IN, USA) for the treatment and control of immature adult and adult *T. canis* and adult *T. leonina* in dogs.

## Methods

All studies were conducted according to VICH GL9 Good Clinical Practice, VICH GL19 Effectiveness of Anthelmintics: Specific Recommendations for Canines, VICH GL7 Effectiveness of Anthelmintics: General Recommendations, and World Association for the Advancement of Veterinary Parasitology (WAAVP): Second edition of guidelines for evaluating the efficacy of anthelmintics for dogs and cats [19, 20]. The protocols were reviewed and approved by the study site’s Animal Care and Use Committee and by the Elanco Animal Health Care and Use Committee prior to initiation.

A total of six studies were conducted to provide substantial evidence to the US FDA Center for Veterinary Medicine for the treatment and control of *T. canis* and *T. leonina*, evaluating efficacy and safety against immature adult and adult infections of *T. canis* and adult infections of *T. leonina.* An overview of the parasite stages targeted, inoculation day, number of infective units administered, and treatment and necropsy days for each study is presented in Table 1. Studies 4 and 6 utilized naturally infected animals, while all remaining studies utilized experimental inoculations.

**Table 1:** Overview of study designs evaluating efficacy of Credelio Quattro against *Toxocara canis* and *Toxascaris leonina*

Study no.	Parasite	Isolate origin	Stage	*N*	Inoculum (L_3_ larvated eggs) administered	Inoculation study day	Treatment study day	Necropsy study day
1	*T. canis*	USA	Immature adult	10/group	300	−24	0	7
2	*T. canis*	EU	Immature adult	10/group	300	−24	0	7
3	*T. canis*	USA	Adult	10/group	300	−48	0	10
4	*T. canis*	MAR	Adult	> 10/group	NA	Natural infection	0	10
5	*T. leonina*	USA	Adult	10/group	500	−63	0	10
6	*T. leonina*	MAR	Adult	> 10/group	NA	Natural infection	0	10

*EU* Europe, *MAR* Morocco, *USA* United States of America

In the natural infection studies (Studies 4 and 6), it was anticipated that a number of dogs screened for naturally acquired gastrointestinal nematode infections would test positive for mixed nematode infections. To respect 3Rs (Replacement, Reduction, and Refinement) and to utilize all the information provided by an individual dog, a central randomization plan shared between the natural infection studies was used to allow dogs to be included in each of the studies, where they tested positive for the intended parasite (*Toxocara canis*, *Toxascaris leonina*), subject to the specific criteria of the individual study. Although this created uneven treatment groups, and more than 20 dogs were used per study, some dogs participated in both studies, minimizing the overall number of dogs required.

### Animals

All studies enrolled male and female Beagle or crossbred dogs, either intact or altered, confirmed to be in good health by physical examination. Dogs were ≤ 14 weeks of age at the time of experimental inoculation in Studies 1–3 and 5. In Studies 4 and 6 utilizing naturally infected animals, dogs were between 3 months and 10 years of age at the time of treatment administration. To qualify for enrollment, dogs were required to be in good health with no evidence of serious disease, neither pregnant nor lactating, weigh more than 3.7 kg at the time of dosing, and confirmed positive for the presence of *T. canis*/*T. leonina* eggs. Dogs knowingly exposed to an anthelmintic or endectocide with activity against roundworms within the expected period of potential persistence prior to experimental inoculation or treatment administration, or dogs that tested positive for the presence of roundworm eggs prior to experimental inoculation, were excluded.

Study animals were fed an age-appropriate standard commercially available diet and were provided potable water ad libitum. Dogs in Studies 4 and 6 were individually housed throughout the duration of the study, while dogs in all remaining studies were pair-housed until allocation to treatment groups and individual housing. Enrichment items were available to all study animals at all times. Temperature and humidity were adequately controlled, and a photoperiod of 12-h light and 12-h dark was maintained throughout all studies with the exception of Studies 4 and 6, in which facilities were designed to allow natural light.

### Randomization and treatment

Prior to treatment, dogs meeting the inclusion requirements and none of the exclusion requirements were randomized according to a complete randomization design to receive either a control product (CP) with vehicle control or the investigational veterinary product (IVP) Credelio Quattro containing 20–40 mg/kg lotilaner, 0.02–0.04 mg/kg moxidectin, 5–10 mg/kg praziquantel, and 5–10 mg/kg pyrantel (as pamoate salt). On the basis of pre-treatment body weight obtained on Day −1, dogs received the appropriate combination of tablets to provide as close to the minimum effective dosages of 20.0 mg/kg lotilaner, 0.02 mg/kg moxidectin, 5.0 mg/kg praziquantel, and 5.0 mg/kg pyrantel as possible, without underdosing. Treatment with CP and IVP was administered orally to dogs in a fed state.

### Safety assessments

All study animals were observed once daily prior to treatment and then at least twice daily after treatment, continuing throughout the in-life phase of each study. On the day of dosing, animals were evaluated prior to treatment administration as well as 1-, 2-, 4-, and 8-h post-treatment. Any abnormal observations reported after the administration of an IVP, whether or not considered to be product-related, were recorded as adverse health events. No veterinary drugs or therapies other than protocol-specific treatments were administered unless the investigator or veterinarian deemed medical treatment necessary. Administration of all concomitant medications was documented. Dogs were not exposed to any anthelmintics or endectocides that could impact the results of the study. In the experimentally induced studies, due to the young age, puppies were administered antiprotozoals such as ponazuril for treatment of coccidiosis and, in Study 5, the dogs received fenbendazole for the treatment of *Giardia*, a week prior to beginning acclimation.

### Experimental inoculation

Studies 1–3 and 5 utilized experimental inoculations with either *T. canis* or *T. leonina* larvated eggs according to study designs outlined in Table 1. Roundworm eggs were recovered from adult female worms, cultured in vitro until larvated and then administered orally to study dogs. Animals were inoculated orally with a single dose of larvated *T. canis* eggs 24 days prior to treatment to evaluate efficacy against immature adult stages (Studies 1 and 2) and 48 days prior to treatment to evaluate efficacy against adult stages (Study 3). For *T. leonina*, animals were orally inoculated with larvated eggs 63 days prior to treatment to evaluate efficacy against adult stages (Study 5).

All isolates utilized for experimental inoculation were obtained from the field less than 10 years from the time of each inoculation. Studies 1 and 3 utilized *T. canis* isolates obtained from naturally infected dogs from Georgia (2021) and Tennessee (2020), respectively. Study 2 used an isolate obtained from Italy (2016). Study 5 used a *T. leonina* isolate obtained from a naturally infected dog in Montana (2022). Isolates were maintained by inoculation of donor dogs as required. Studies 4 and 6 utilized dogs naturally infected with *T. canis* and *T. leonina* from Morocco.

### Fecal egg counts

Prior to inoculation in experimental induced studies, fecal samples (freshly voided or obtained directly from the rectum) were collected from each candidate, processed using a centrifugation-flotation technique, and qualitatively examined to confirm the absence of any pre-existing gastrointestinal nematode infections. In Studies 3–6, a quantitative fecal exam was conducted on each dog to calculate fecal egg count (FEC), pre- and post-treatment, to evaluate the percent reduction in FEC after administration of the novel combination tablet. Studies 3 and 5 utilized Modified Wisconsin and Studies 4 and 6 utilized McMaster. Briefly, for Modified Wisconsin, 1 g of feces was mixed with flotation solution (sugar, ~1.2 s.g.), strained into a 15 mL centrifuge tube, filling the tube within approximately 2–3 mm of the top and centrifuged at ~500*g* for 5 min. After centrifugation, additional flotation solution was added to samples until a slight meniscus formed. A coverslip was placed on the centrifuge tube and allowed to sit for 10 min before examination. For McMaster, 2 g feces were mixed with 58 mL flotation solution (sugar) and then pipetted into McMaster slide chambers. Slides were allowed to sit for 5 min before examination.

### Necropsy and worm counts

All enrolled dogs were euthanized 7- or 10-days post-treatment according to the American Veterinary Medicine Association guidelines for the euthanasia of animals, and postmortem specimens were processed for recovery of gastrointestinal parasites. Prior to necropsy, dogs were fasted overnight. During necropsy, the abdominal cavity was opened and examined for abnormalities, and the digestive tract was ligated. The entire gastrointestinal tract from stomach to rectum was removed and placed into separate labeled containers. Once removed, the gastrointestinal tract was split longitudinally, and the mucosal surfaces were scraped and rinsed with water to remove contents and parasites. Gut contents and scrapings were washed over a sieve [no. 100 (150 µm) for immature adult, no. 60 (250 µm) for adult ascarids], preserved in 10% formalin solution, and examined for parasite recovery, identification, and enumeration. Intact specimens were identified by gender and enumerated separately. When worm fragments were recovered, final worm counts were based on the total number of heads or tails, whichever was greater. Total worm counts and gender were documented.

### Statistical analysis

Statistical analyses were performed utilizing SAS version 9.4 (SAS Institute, Cary, NC, USA). All hypotheses were tested using two-sided tests at the 0.05 level of significance. The experimental unit for all studies reported here was the individual dog. A minimum of five worms needed to be present in at least six control dogs to show an adequate infection in the control group. Efficacy was determined post-treatment by comparison of the total *T. canis* worm count (Studies 1–4), or total *T. leonina* worm count (Studies 5 and 6) in the treated group versus the control group. A logarithmic transformation (ln[count + 1]) was applied to the post-treatment *T. canis* and *T. leonina* worm counts for each individual animal to address the skewed nature of the data and stabilize the variance. The transformed counts were analyzed using an analysis of variance (ANOVA) model with a fixed effect of treatment, and the natural infection Studies 4 and 6 also included a random effect for cohort. Each treated group was compared with the control group in a separate statistical model. Geometric mean (GM) worm counts were estimated by back-transforming model Least Squares (LS) means. In addition, for studies that evaluated efficacy against adult *T. canis* and *T. leonina* (Studies 3–6), the GM fecal eggs per gram (EPG) pre-treatment and 10 days post-treatment were calculated, and the percent FEC reduction was reported for each treatment group.

## Results

The most common adverse events reported, which occurred in both control- and treated-groups and are commonly reported in dogs with gastrointestinal parasite infections, included digestive tract disorders such as diarrhea, mucus and/or blood in feces, vomiting, and expelled ascarid worms. Adequacy of infection, defined by VICH and WAAVP as at least six control animals with a minimum of five parasites collected at necropsy, was achieved in all studies reported [19, 20]. GM worm counts and percent efficacy for each study are reported in Table 2 for all *T. canis* studies and in Table 3 for all *T. leonina* studies. Pre- and post-treatment GM FECs and percent reduction for Studies 3–6 are reported in Table 4 for both species.

**Table 2:** Efficacy of a single oral dose of Credelio Quattro against *Toxocara canis* infections in dogs

Study no.	Treatment group	Stage	*N*	Geometric mean worm count (range)	% efficacy	Statistical comparison with control group (test statistic)
1	Control	Immature adult	10	9.4 (0–45)	–	–
1	Credelio Quattro	Immature adult	10	0.2 (0–2)	97.9	*P* < 0.0001 (*t*_18_ = 5.25)
2	Control	Immature adult	10	6.0 (0–43)	–	–
2	Credelio Quattro	Immature adult	10	0.0 (0–0)	100	*P* < 0.0001 (*t*_18_ = 5.26)
3	Control	Adult	10	7.7 (2–18)	–	–
3	Credelio Quattro	Adult	10	0.2 (0–1)	97.0	*P* < 0.0001 (*t*_18_ = 9.29)
4	Control	Adult (natural infection)	14	4.3 (0–36)	–	*–*
4	Credelio Quattro	Adult (natural infection)	13	0.04^a^ (0–0)	99.0	*P* < 0.0001 (*t*_20_ = 5.51)

^a^ No *T. canis* worms were observed in any dog in the treated group; however, the statistical model estimate of GM was 0.04

**Table 3:** Efficacy of a single oral dose of Credelio Quattro against *Toxascaris leonina* infections in dogs

Study no.	Treatment group	Stage	*N*	Geometric mean worm count (range)	% efficacy	Statistical comparison with control group
5	Control	Adult	10	17.2 (4–62)	–	*–*
5	Credelio Quattro	Adult	10	0.0 (0–0)	100	*P* < 0.0001 (*t*_18_ = 12.18)
6	Control	Adult (natural infection)	12	5.7 (0–18)	–	–
6	Credelio Quattro	Adult (natural infection)	10	0.0 (0–0)	100	*P* < 0.0001 (*t*_15_ = 7.21)

**Table 4:** Fecal egg count reduction after a single oral dose of Credelio Quattro in dogs

Study no.	Parasite	Treatment group	*N*	Geometric mean fecal egg count (EPG)		% reduction
				Pretreatment mean (range)	Posttreatment mean (range)	
3	*T. canis*	Control	10	190.7 (22–924)	88.1 (0–636)	–
3	*T. canis*	Credelio Quattro	10	121.0 (41–262)	1.5 (0–24)	98.8
4	*T. canis*	Control	14	212.1 (33.3–2400)	91.4 (0–2400)	–
4	*T. canis*	Credelio Quattro	13	200.2 (33.3–1533.3)	0.0 (0–0)	100
5	*T. leonina*	Control	10	309.1 (87.7–858.3)	156.9 (20–650)	–
5	*T. leonina*	Credelio Quattro	10	110.6 (12.3–257)	0.0 (0–0)	100
6	*T. leonina*	Control	12	283.1 (100–1033.3)	205.8 (0–1000)	–
6	*T. leonina*	Credelio Quattro	10	172.4 (66.7–933.3)	0.0 (0–0)	100

*EPG* eggs per gram

### *Toxocara canis*

Credelio Quattro was 97.9% and 100% effective against *T. canis* immature adults (Studies 1 and 2, Table 2). Dogs treated with Credelio Quattro had significantly lower GM worm counts: 0.2 (range 0–2) in Study 1 and 0.0 (range 0–0) in Study 2. Two studies evaluated the efficacy of Credelio Quattro against *T. canis* adult infections (Studies 3 and 4, Table 2), resulting in 97.0% and 99.0% efficacy. In Study 4, which evaluated naturally occurring infections, no worms were recovered from treated dogs. However, the LS means estimated by the statistical model adjusted for cohort differences and resulted in estimated GM worm counts of 0.04 (range 0–0) for the treated group, despite zero observed worms in treated dogs. In Studies 3 and 4 (Table 4), FECs performed 10 days post-treatment demonstrated reductions of 98.8% and 100%, respectively.

### *Toxascaris leonina*

Credelio Quattro was 100% effective against adult stages of *T. leonina* in both experimentally induced and naturally infected animals (Table 3). Percent FEC reduction was demonstrated to be 100% 10 days post-treatment (Table 4) in both studies.

## Discussion

Development of a convenient oral combination product for dogs is important to help increase administration compliance, especially considering the zoonotic risk of *T. canis*, the ability for ascarid eggs to survive for long periods of time in the environment, and the global prevalence of these parasites. Credelio Quattro is the only FDA-approved product containing four active ingredients (lotilaner, moxidectin, praziquantel, and pyrantel) designed to provide robust efficacy against the broadest range of parasites in an isoxazoline endectocide. Providing convenient combination products increases in-clinic purchase compliance as compared with the combination of separate ectoparasitic and endoparasitic products [21].

The dosage of pyrantel in Credelio Quattro is based on the well-established minimum effective dosage rate of 5.0 mg/kg [22]. Efficacy of Credelio Quattro against adult *T. canis* (≥ 97%) and *T. leonina* (100%) presented here highlights the sustained efficacy of pyrantel against ascarids, which remains comparable to its performance over the last three decades.

To minimize environmental contamination, prompt removal of feces combined with the reduction of eggs shed in feces plays a critical role in efforts to control gastrointestinal parasites. Administration of Credelio Quattro provided 98.8% and 100% FEC reduction in *T. canis*-infected dogs and 100% FEC reduction in *T. leonina*-infected dogs, 10 days post-treatment. Reduction of the total number of eggs shed in feces reduces continued environmental contamination of these hardy life stages and helps prevent transmission to other animals, reinfection of animal hosts, and potential zoonotic transmission to humans. In addition, treatment of active infections through the elimination of adult and immature adult stages plays a critical role in parasite control measures. Providing efficacy against immature adult stages reduces the number of worms able to develop to sexually mature adults shedding eggs in the environment. Administration of Credelio Quattro against immature adult *T. canis* in experimentally induced dogs demonstrated 97.9% and 100% efficacy, with a total of only three immature adult worms from all treated dogs recovered at necropsy.

Credelio Quattro demonstrated effectiveness against immature and adult *T. canis* (≥ 97.0%) and adult *T. leonina* (100%) in laboratory studies. These results are in line with the efficacy observed for a comparable endectocide, Simparica Trio^®^ (Zoetis, Parsippany, NJ, USA) containing sarolaner, moxidectin, and pyrantel. Results for Simparica Trio were similar against immature and adult *T. canis* and adult *T. leonina* (≥ 95.2% and ≥ 89.7%, respectively) [23]. The robust efficacy demonstrated in these described laboratory studies is also consistent with recent field efficacy data documented in dogs infected with gastrointestinal nematodes. In this US multi-center field study, Credelio Quattro was evaluated against a positive control product, Simparica Trio. FEC reduction 10 days post-treatment was calculated in *T. canis*-infected dogs, with Simparica Trio providing 96.6% reduction and Credelio Quattro providing 98.7% reduction [24].

## Conclusions

These laboratory studies with experimentally induced and naturally infected animals confirm the effectiveness and safety of a single dose of Credelio Quattro, a novel oral combination chewable tablet administered at the minimum dosages of 20 mg/kg lotilaner, 0.02 mg/kg moxidectin, 5 mg/kg praziquantel, and 5 mg/kg pyrantel for the treatment and control of immature adult and adult *T. canis* and adult *T. leonina* in dogs.

## Data Availability

Data supporting the conclusions of this article are included within the article.

## Abbreviations

ANOVA: Analysis of variance
CAPC: Companion Animal Parasite Council
CP: Control product
EPG: Eggs per gram
FEC: Fecal egg count
FDA: Food and Drug Administration
GM: Geometric mean
IVP: Investigational veterinary product
LS: Least Squares
USA: United States of America
EU: Europe
MAR: Morocco
WAAVP: World Association for the Advancement of Parasitology
